# Automethylation of SUV39H2, an oncogenic histone lysine methyltransferase, regulates its binding affinity to substrate proteins

**DOI:** 10.18632/oncotarget.8072

**Published:** 2016-03-14

**Authors:** Lianhua Piao, Makoto Nakakido, Takehiro Suzuki, Naoshi Dohmae, Yusuke Nakamura, Ryuji Hamamoto

**Affiliations:** ^1^ Section of Hematology/Oncology, Department of Medicine, The University of Chicago, Chicago, IL 60637, USA; ^2^ Biomolecular Characterization Unit, RIKEN Center for Sustainable Resource Science, Wako, Saitama 351-0198, Japan; ^3^ Division of Molecular Modification and Cancer Biology, National Cancer Center, Chuo-ku, Tokyo 104-0045, Japan

**Keywords:** SUV39H2, oncogene, automethylation, SET domain

## Abstract

We previously reported that the histone lysine methyltransferase SUV39H2, which is overexpressed in various types of human cancer, plays a critical role in the DNA repair after double strand breakage, and possesses oncogenic activity. Although its biological significance in tumorigenesis has been elucidated, the regulatory mechanism of SUV39H2 activity through post-translational modification is not well known. In this study, we demonstrate *in vitro* and *in vivo* automethylation of SUV39H2 at lysine 392. Automethylation of SUV39H2 led to impairment of its binding affinity to substrate proteins such as histone H3 and LSD1. Furthermore, we observed that hyper-automethylated SUV39H2 reduced methylation activities to substrates through affecting the binding affinity to substrate proteins. Our finding unveils a novel autoregulatory mechanism of SUV39H2 through lysine automethylation.

## INTRODUCTION

Histone methylation plays important key roles in the regulation of gene transcription, genome integrity and epigenetic inheritance [1–3]. Histones can be methylated on lysine and arginine residues, and in general, histone methyltransferases, including histone lysine methyltransferases (HKMTs) and protein arginine methyltransferases (PRMTs), are the main enzymes catalyzing the transfer of methyl groups from the cofactor S-adenosyl methionine (SAM) to lysine and arginine residues [4–6]. In addition to histone proteins, recent findings have indicated that histone methyltransferases are involved in multiple cellular functions through targeting a large number of non-histone substrate proteins [7–13]. The Su(var) 3-9, Enhancer-of-zeste, Trithorax (SET) domain-containing proteins constitute the major group of HKMTs. SET domain-containing proteins are characterized by the SET domain consisting an approximately 130-140 amino acid, evolutionary well conserved sequence motif, which was initially discovered in the Drosophila proteins Su(var) 3-9, Enhancer-of-zeste and Trithorax [14]. In addition to the SET domain, SUV39 family proteins possess pre-SET (N-SET) and post-SET (C-SET) domains at the amino and carboxyl boundaries of the SET domain respectively [15, 16]. Unlike the pre-SET and SET domain, the post-SET domain does not have some specific structure, but is proximal to the active site [17]. The post-SET domain includes three conserved cysteine residues that are reported to be crucial for HKMT activity in the SUV39 family proteins. Substitution of these cysteine residues completely abolished the enzymatic activity of DIM-5 and SETDB1 [18, 19]. SUV39H1 lacking the post-SET domains also lost detectable HKMT activity [20].

Suppressor of Variegation 3-9 Homologue 2 (SUV39H2, also known as KMT1B), one of the histone lysine-specific methyltransferases, could selectively methylate lysine 9 of histone H3 (H3K9), which is associated with heterochromatin formation and transcriptional repression [21]. *Suv39h*-deficient mice exhibit severe impaired viability and chromosomal instabilities [22]. The primary cells derived from *Suv39h*-double-null (*Suv39h-*dn*)* mice exhibit abnormally long telomeres with reduced binding to the chromobox proteins Cbx1, Cbx3 and Cbx5 [23], and knockdown of SUV39H1 and SUV39H2 also showed increasing telomere length in pigs [24]. We previously reported that SUV39H2 was overexpressed in various types of cancer, while its expression is hardly detectable in normal tissues except testis [25, 26]. In addition, SUV39H2-dependent histone H2AX methylation directly affects the level of γ-H2AX activity that regulates the DNA repair pathway in human cancer, and we confirmed that SUV39H2 possesses oncogenic activity [26]. Recently, we also reported that SUV39H2-mediated methylation of LSD1 at Lys 322 inhibits its polyubiquitination, and leads to stabilization of the LSD1 protein in human [27]. Although SUV39H2 is considered as one of the promising targets for anti-cancer drug development, the regulatory mechanism of SUV39H2 activity by post-translational modification is still not well known. Hence, it is important to elucidate the detailed mechanism to proceed with anti-cancer drug development efficiently. In the present study, we describe the automethylation of SUV39H2 at lysine 392, and this automethylation regulates its binding affinity to SUV39H2-substrate proteins.

## RESULTS

### SUV39H2 is automethylated at lysine 392 *in vitro*

**Figure 1:**
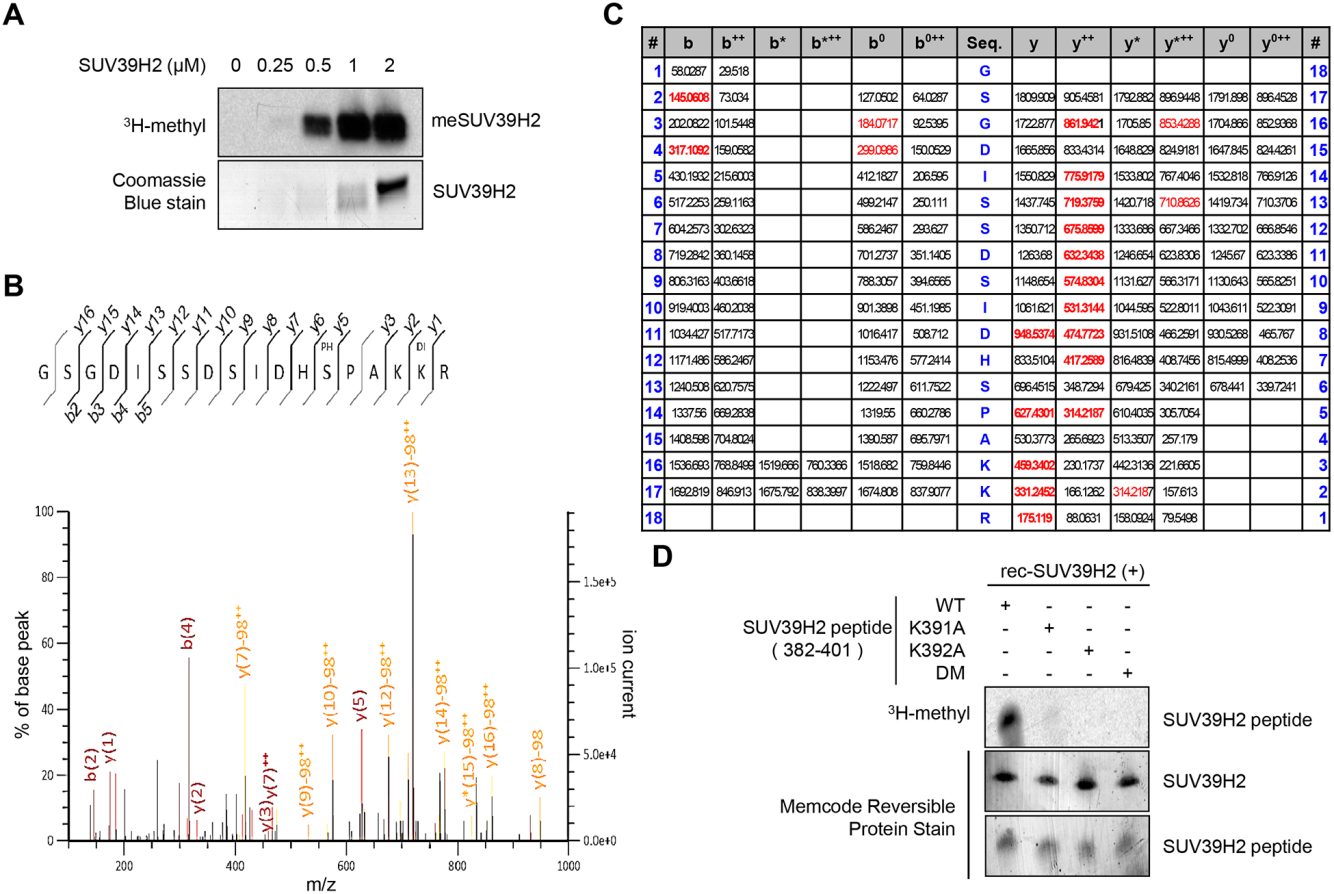
Lysine 392 on SUV39H2 is automethylated **A.** The automethylation intensities of SUV39H2 were proportional to the amounts of SUV39H2 protein. An *in vitro* methyltransferase assay was performed by using purified His-tagged SUV39H2 recombinant proteins. Methylated SUV39H2 was detected by autoradiography. Amounts of loading proteins were confirmed by staining with Coomassie Brilliant Blue. **B.** The MS-MS spectrum corresponding to the dimethylated and phosphorylated SUV39H2 376–393 peptide. The 28 Da increase of the lysine 392 residue was detected. **C.** MS/MS spectra of the SUV39H2 peptide corresponding to 376-393 amino acids. LC-MS/MS analysis showed methylation of SUV39H2 at lysine 392. Theoretical values of MS fragments are summarized. **D.** An *in vitro* methyltransferase assay indicated that wild-type SUV39H2 peptide (WT, 382-401 amino acids) was methylated by His-tagged SUV39H2 recombinant proteins but not lysine 391, lysine 392, or both lysines 391 and 392-substituted SUV39H2 peptide (K391A/K392A/DM). Each SUV39H2 peptide (WT, K391A, K392A or K391A/K392A/DM) was mixed with His-tagged recombinant protein in 50 mM Tris-HCl (pH8.8) buffer with 1.0 μCi/ml S-adenosyl-L-[mehyl-^3^H]-methionine for 2 hours at 30°C. After boiling in the sample buffer, the samples were subjected to SDS-PAGE, and visualized by autoradiography. Amounts of loading proteins were evaluated by staining the MemCode™ Reversible Protein Stain (Thermo Fisher Scientific).

**Figure 2:**
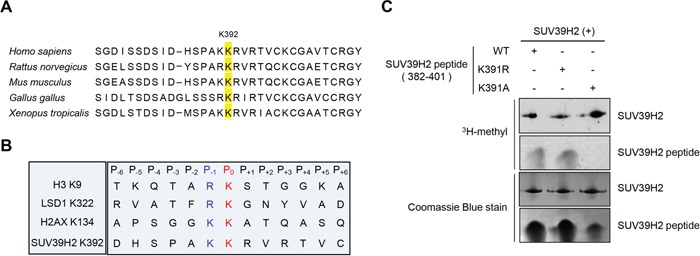
K/R-K sequence is important for SUV39H2-mediated methylation **A.** Amino acid sequence indicated that the methylation site lysine 392 of SUV39H2 was highly conserved across species. **B.** Sequence alignment of the methylation sites of the three known substrates and automethylation site. Lysines at P_0_ position were the methylation sites (highlighted in red). Amino acids at P_-1_ position were highlighted in blue. **C.** Both wild-type SUV39H2 peptide (382-401 amino acids, WT) and K391R peptide were methylated by His-tagged SUV39H2 recombinant proteins, whereas K391A peptide was not methylated. Each SUV39H2 peptide (WT, K391R or K391A) was mixed with His-tagged recombinant protein in 50 mM Tris-HCl (pH8.8) buffer with 1.0 μCi/ml S-adenosyl-L-[mehyl-^3^H]-methionine for 2 hours at 30°C. After boiling in the sample buffer, the samples were subjected to SDS-PAGE, and visualized by autoradiography. Amounts of loading proteins were confirmed by staining with Coomassie Brilliant Blue.

In our previous study, automethylation of SUV39H2 has been observed when we performed *in vitro* methyltransferase assays using recombinant SUV39H2 protein as the enzyme source. The automethylation intensities of SUV39H2 were proportional to the amounts of SUV39H2 protein (Figure 1A). Subsequent analysis by liquid chromatography-tandem mass spectrometry (LC-MS/MS) identified lysine 392 was automethylated (Figure 1B and 1C). To verify this automethylation site, an *in vitro* methyltransferase assay with S-adenosyl-L-[^3^H-methyl]-methionine as a cofactor was conducted using synthesized three peptides corresponding to codons 382-401 of SUV39H2, one possessing the wild-type sequence (WT) and the other two peptides having substitution of lysine 391 to alanine (K391A) or lysine 392 to alanine (K392A). An intense methylation signal was detected for the WT peptide while no methylation signal was detected when the two substituted peptides were used as substrates (Figure 1D). This result implies that not only lysine 392 but also lysine 391 is critical for automethylation activity. Sequence alignment showed that lysine 392 (an automethylation site indicated by LC-MS/MS) is highly evolutionary conserved among various species (Figure 2A), supporting the importance of this lysine residue. Alignment of amino acid sequences of three known substrate proteins methylated by SUV39H2 [20, 26, 27] suggests “K/R-K” as a possible consensus motif for methylation by SUV39H2 (Figure 2B), and P_-1_ position residues of SUV39H2 are either lysine or arginine residue among multiple species (Figure 2A). Consistent with this conserved “K/R-K” motif, we observed a significant reduction of methylation signal in the peptide in which lysine 391 was substituted with alanine (Figure 1D). Given these results, we hypothesize the lysine or arginine residue at P_-1_ position appears to be essential for SUV39H2 recognition of a P_0_ targeted lysine residue. To examine this hypothesis, we synthesized a peptide in which lysine 391 was substituted with an arginine residue and compared methylation signals among wild-type, K391R-substituted and K391A-substituted SUV39H2 peptides. As shown in Figure 2C, WT and K391R SUV39H2 peptides revealed the similar intensity of methylation signals, whereas the methylation signal was not observed in the K391A peptide. This result further supports importance of the P_-1_ position of the P_0_ targeted lysine residue for methylation by SUV39H2.

### *In vivo* automethylation of SUV39H2 at lysine 392

**Figure 3:**
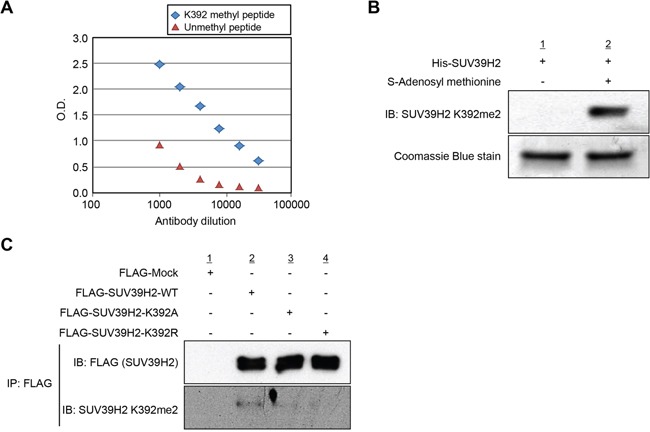
Automethylation of SUV39H2 is validated *in vivo* **A.** Determination of the titer and specificity of the anti-K392 dimethylated SUV39H2 (Sigma-Aldrich) antibody analyzed by enzyme-linked immunosorbent assay (ELISA). A constant amount of K392 methyl peptide or unmethyl peptide has been coated into the wells of the ELISA, and tested with different dilutions of the antibody. **B.** His-tagged SUV39H2 recombinant proteins were incubated with or without the cofactor SAM at 30°C for 2 hours. Automethylated SUV39H2 protein was blotted with the anti-SUV39H2 K392me2 antibody, and amounts of loading SUV39H2 recombinant proteins were measured by staining with Coomassie Brilliant Blue. **C.** *In vivo* methyltransferase experiment was conducted in 293T cells overexpressing FLAG control empty vector (FLAG-Mock), FLAG-tagged SUV39H2 wild-type (FLAG-SUV39H2-WT), FLAG-tagged SUV39H2 K392A mutant (FLAG-SUV39H2-K392A) or FLAG-tagged SUV39H2 K392R mutant (FLAG-SUV39H2-K293R). Cells were lysed with RIPA buffer 48 hours after transfection, and samples were immunoblotted with anti-FLAG and anti-SUV39H2 K392me2 antibodies.

To examine the automethylation of SUV39H2 *in vivo*, we generated an anti-K392 dimethylated SUV39H2 antibody and confirmed the specificity of this antibody against K392 dimethylated peptide by enzyme-linked immunosolvent assay (ELISA) (Figure 3A). We further validated this methylation-specific antibody by *in vitro* methyltransferase assay as shown in Figure 3B and confirmed that this antibody could detect recombinant SUV39H2 proteins in the presence of the methyl donor SAM but not in the absence of SAM. Using this methylation-specific antibody, we subsequently performed western blot analysis after immunoprecipitation experiments. 293T cells were transfected with the plasmid designed to express either FLAG-tagged wild-type SUV39H2 (WT), K392A SUV39H2 (lysine 392 was substituted by alanine) or K392R SUV39H2 (lysine 392 was substituted by arginine). As shown in Figure 3C, a methylation signal was detected in FLAG-SUV39H2-WT, but not in FLAG-SUV39H2-K392A or FLAG-SUV39H2-K392R, which confirms automethylation of SUV39H2 at lysine 392 *in vivo*. Furthermore, the human lung adenocarcinoma A549 cells and the human small cell lung cancer SBC-5 cells were treated with chaetocin, an inhibitor for methyltransferase activity of the SU(VAR) 3-9, and we confirmed that automethylation of SUV39H2 at lysine 392 was diminished after treatment with chaetocin (Supplementary Figure S1), implying that automethylation of endogenous SUV39H2 at lysine 392 is also observed in cancer cells.

### Automethylation diminishes binding affinity of SUV39H2 to substrate proteins

**Figure 4:**
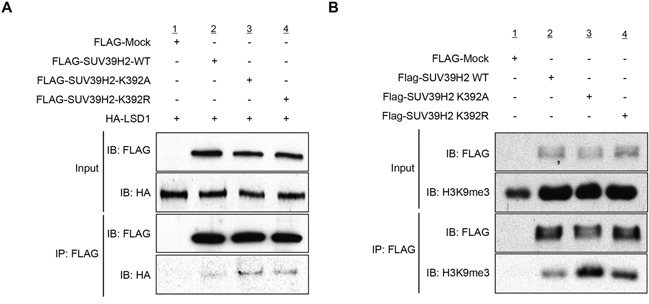
Automethylation of SUV39H2 blocks the protein-substrate interaction **A.** 293T cells were co-expressed with HA-tagged LSD1 and FLAG-tagged SUV39H2-WT, SUV39H2-K392A or SUV39H2-K392R. After 48 hours of incubation, cells were lysed with RIPA buffer, followed by immunoprecipitation with anti-FLAG M2 affinity gel. Immunoprecipitates were immunoblotted with anti-FLAG and anti-HA antibodies. **B.** 293T cells were transfected with FLAG-tagged SUV39H2-WT, SUV39H2-K392A or SUV39H2-K392R. Interaction of endogenous histone H3 and exogenous SUV39H2 proteins was examined by western blot analysis.

In order to examine the effect of SUV39H2 automethylation on the interaction with substrate proteins, 293T cells were transfected with HA-LSD1, a substrate of SUV39H2, and FLAG-Mock, FLAG-SUV39H2-WT, FLAG-SUV39H2-K392A or FLAG-SUV39H2-K392R. After 48 hours of incubation, cells were lysed with RIPA buffer, followed by immunoprecipitation using anti-FLAG M2 affinity gel. Then, immunoprecipitates were immunoblotted with anti-FLAG and anti-HA antibodies. As shown in Figure 4A, FLAG-SUV39H2-K392A and FLAG-SUV39H2-K392R showed higher affinity to HA-LSD1 than FLAG-SUV39H2-WT, suggesting that SUV39H2 automethylation at lysine 392 appears to decrease the binding affinity of SUV39H2 to LSD1. To verify this result, we transfected FLAG-SUV39H2-WT, FLAG-SUV39H2-K392A or FLAG-SUV39H2-K392R into 293T cells to examine the effect of SUV39H2 automethylation on the interaction with histone H3, which is also a substrate of SUV39H2. After 48 hours of incubation, cells were lysed with RIPA buffer, followed by immunoprecipitation using anti-FLAG M2 affinity gel. Subsequently, immunoprecipitated samples were immunoblotted with anti-FLAG and anti-histone H3 antibodies. As shown in Figure 4B, FLAG-SUV39H2-K392A or FLAG-SUV39H2-K392A showed higher affinity to histone H3 than FLAG-SUV39H2-WT, and this is concordant with the LSD1 result. Taken together, these data indicate that automethylation diminishes binding affinity of SUV39H2 to substrate proteins.

### Automethylation regulates the function of SUV39H2 through controlling the binding affinity to substrate proteins

**Figure 5:**
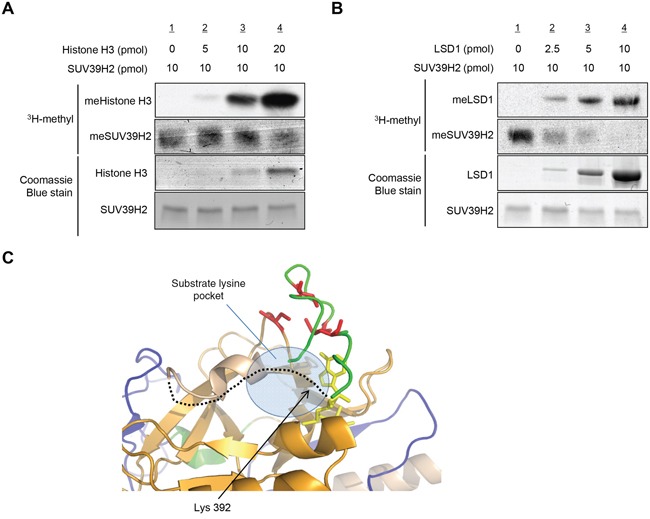
Automethylation of SUV39H2 and methylation of substrate proteins are exclusively correlated **A-B.** Recombinant SUV39H2 proteins were individually incubated with increasing amount of histone H3 protein (A) or LSD1 protein (B) in the presence of S-adenosyl-L-[methyl-^3^H]-methionine. The reaction mixtures were separated by SDS-PAGE, and the intensities of methylated proteins were detected by autoradiography, whereas the loading amounts of proteins were stained with Coomassie Brilliant Blue. **C.** The automethylation site (lysine 392) is located in a flexible loop (black dot line), which is suggested to form a part of the substrate lysine pocket. Pre-SET, SET and Post-SET domains are colored blue, orange, and green, respectively. SAM is shown as yellow stick and four cysteine residues corresponding to zinc binding are highlighted with red color.

We next conducted an *in vitro* methyltransferase assay of recombinant SUV39H2 using different concentration of recombinant histone H3 as a substrate. Importantly, the automethylation of SUV39H2 was attenuated in the presence of histone H3 in a dose-dependent manner (Figure 5A). Similarly, when we used recombinant LSD1 as a substrate, it also decreased the automethylation of SUV39H2 in a dose-dependent manner (Figure 5B). These results indicate that automethylation levels of SUV39H2 and methylation levels of SUV39H2 substrate proteins appear to be reversely correlated.

**Figure 6:**
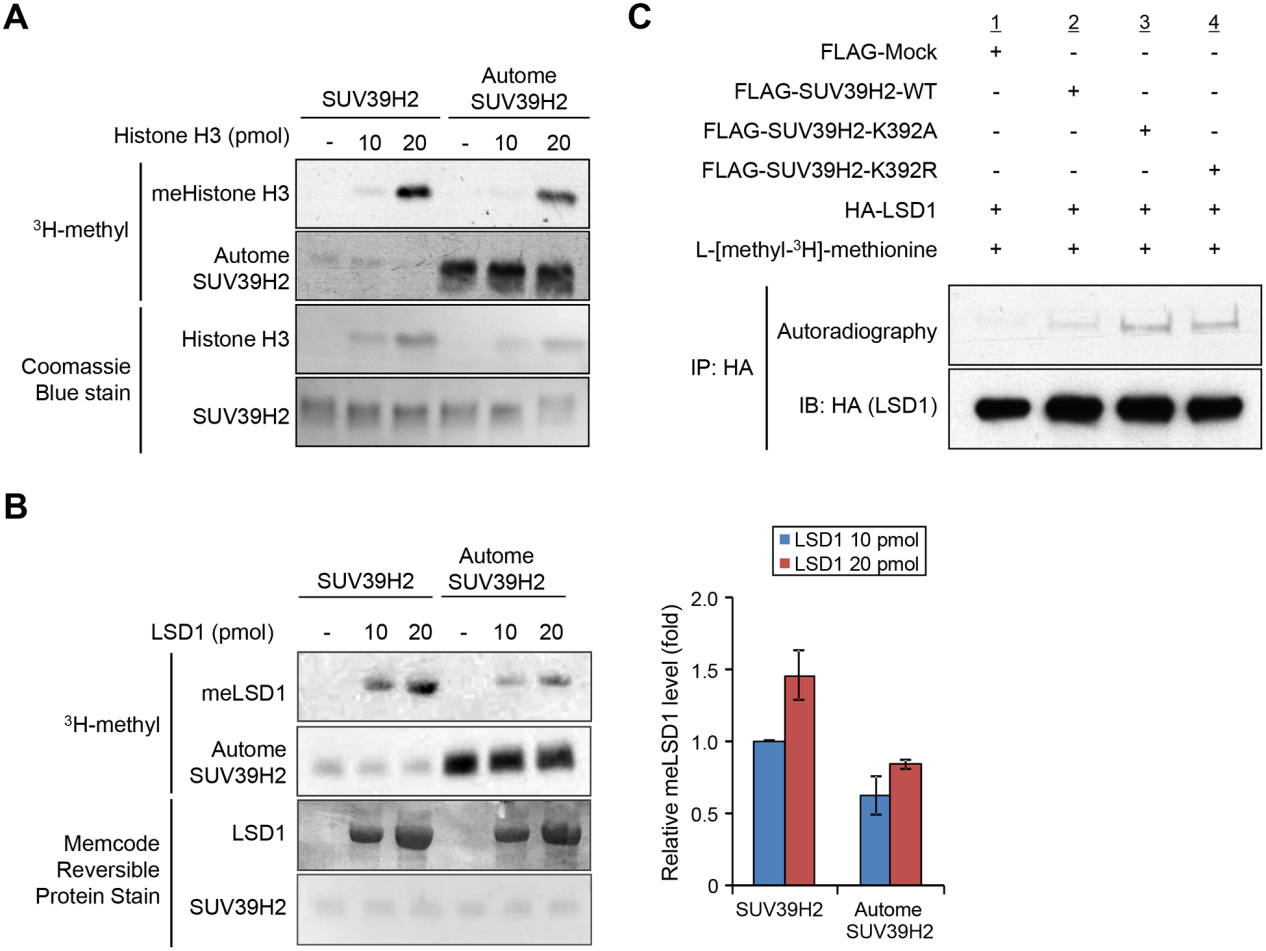
Automethylation of SUV39H2 negatively regulates SUV39H2 methylation activities to other substrates **A-B.** Recombinant H3 protein (A) or recombinant LSD1 protein (B) was reacted with equal amount of recombinant SUV39H2 proteins or hyper-automethylated SUV39H2 (pre-incubated with S-adenosyl-L-[methyl-^3^H]-methionine for 4 hours). The intensity of methylated LSD1 protein for each sample was quantified by GS-800™ calibrated densitometer (Bio-Rad) and plotted (right panel). All error bars indicate SEM of two independent experiments. Values are relative to the intensity obtained from the reaction of 10 pmol LSD1 and hypomethylated SUV39H2. The loading amounts of proteins were stained with Coomassie Brilliant Blue (A) and MemCode™ Reversible Protein Stain (B). **C.** 293T cells co-expressing HA-LSD1 with FLAG-SUV39H2-WT, FLAG-K392A or FLAG-K392R were labelled with L-[methyl-^3^H]-methionine *in vivo*. Cell lysates were immunoprecipitated with anti-HA agarose (Sigma-Aldrich) and methylated LSD1 was visualized by autoradiography. Immunoprecipitates were immunoblotted with anti-HA antibody as an internal control.

The automethylated lysine 392 located within the post-SET domain; recent studies suggest that post-SET domain is relatively flexible or disordered without the cofactor SAM or peptide binding [15, 18, 28]. On the basis of the structures of SUV39 family methyltransferases, it was proposed that closing of the post-SET domain onto the substrate is necessary for forming catalytically competent conformation. This flexibility may play critical roles in the complex formation with substrate and the cofactor SAM (Figure 5C). Indeed, three cysteine residues in the post-SET domain of SUV39 family, which corresponds to cysteine 398, cysteine 400 and cysteine 405 of SUV39H2, were described to be necessary for HKMT activity [17, 19, 20]. These cysteine residues in the post-SET domain and one cysteine from the SET domain coordinate the binding of a fourth zinc ion (ZnCys4) [17, 28, 29], indicating that proper location of these domains would be essential to bind substrates within the binding pocket. In addition, the crystal structure of SUV39H2 implied that a region from aspartic acid 379 to lysine 392, including automethylation site, on SUV39H2 would form a flexible loop (accession code 2R3A) [15]. Given that this flexible loop is a part of the substrate lysine pocket (Figure 5C), we hypothesized that the automethylation at lysine 392 may influence the accessibility of substrates into the pocket. To elucidate this possibility, we conducted *in vitro* methyltransferase assays. Recombinant histone H3 or LSD1 was reacted with SUV39H2 that was not incubated with SAM or SUV39H2 that was pre-incubated with SAM for 4 hours. As shown in Figure 6A and 6B, hyper-automethylated SUV39H2 that was pre-incubated with SAM, showed less ability to transfer methyl groups to substrate proteins compared to SUV39H2 that was not incubated with SAM. Furthermore, to verify this result, we also conducted an *in vivo* labeling experiment. 293T cells were transfected with HA-LSD1 and FLAG-Mock, FLAG-SUV39H2-WT, FLAG-SUV39H2-K392A or FLAG-SUV39H2-K392R. After 48 hours of incubation, cells were incubated in a medium containing L-[methyl-^3^H]-methionine for 3 hours, followed by immunoprecipitation using anti-FLAG M2 affinity gel. Immunoprecipitates were separated by SDS-PAGE and specific signals were detected by autoradiography. As shown in Figure 6C, we observed more methylated LSD1 proteins in the cells overexpressing K392A- or K392R-substituted SUV39H2 than those overexpressing wild-type SUV39H2. Taken together, these results indicate that automethylation of SUV39H2 at lysine 392 regulates the function of SUV39H2 through controlling the binding affinity to substrate proteins.

## DISCUSSION

Increasing evidence indicates that histone methyltransferases are involved in human tumorigenesis and furthermore, plays crucial roles in the development of many types of cancers [30–38]. So far, most of the HKMTs identified harbor a conserved methyltransferase region termed the SET domain [17, 18, 39–41]. Among HKMTs, *Su(var)* genes were initially identified by genetic screens on centromeric position effects in *Drosophila melanogaster* and *Schizosaccharomyces pombe* [42, 43] and characterized family members actually represent enzymes that can modify chromatin [44]. Suv39h2, the murine homologue of human SUV39H2, was then isolated and characterized as the second methyltransferase targeting H3K9, and contributing to heterochromatin formation [21]. Our previous study showed that the expression levels of human SUV39H2 in various types of cancer tissues are significantly elevated compared with normal tissues [25, 26]. In addition, SUV39H2 shows oncogenic activity, and knockdown of SUV39H2 by specific siRNAs suppresses the growth of cancer cells [25, 26], suggesting that SUV39H2 appears to be an ideal target for the development of anti-cancer therapy.

In this study, we identified the automethylation of SUV39H2 at lysine 392 within the post-SET domain, and a consensus methylation motif of SUV39H2, K/R-K, was disclosed. Additionally, unautomethylated SUV39H2 showed higher affinities to substrate proteins such as LSD1 and histone H3. We also observed that hyper-automethylated SUV39H2 reduced methyltransferase activities to substrates, probably because they reduced the binding affinity to SUV39H2. Hence, we propose that SUV39H2 may regulate interactions and dissociations of substrates and a cofactor through automethylation of SUV39H2 at lysine 392. So far, automethylation of G9a, a histone lysine methyltransferase, was reported; automethylation affects substrate specificity and HP1 binding [45]. However, the physiological and biological functions of lysine automethylation remained almost unknown. Here, we unveil a novel mechanism that lysine automethylation regulates the function of SUV39H2 through controlling its binding affinity to substrate proteins. Since we confirmed that lysine automethylation was frequently observed in a number of histone lysine methyltransferases, further functional analyses may explore physiological and biological importance of this modification.

## MATERIALS AND METHODS

### Cell culture

293T and A549 cells were obtained from American Type Culture Collection (ATCC, Manassas, VA) and SBC-5 cells were obtained from Japanese Collection of Research Bioresources (JCRB, Ibaraki, Japan). These cells were tested and authenticated by DNA profiling for polymorphic short tandem repeat (STR) markers (Supplementary Table S1). All cell lines were grown in monolayers in appropriate media: Dulbecco's modified Eagle's medium (D-MEM) for 293T cells; RPMI1640 medium for A549 cells; Eagle's Minimum Essential Medium (E-MEM) for SBC5 cells supplemented with 10% fetal bovine serum and 1% antibiotic/antimycotic solution (Sigma-Aldrich, St. Louis, MO).

### Antibodies

The following primary antibodies were used: anti-FLAG (rabbit, F-7425; Sigma-Aldrich; dilution used in WB: 1:8000), anti-HA (Y-11) (rabbit, sc-805; Santa Cruz Biotechnology, Santa Cruz, CA; dilution used in WB: 1:2000), anti-H3K9me3 (rabbit, ab8898; Abcam, Cambridge, UK; dilution used in WB: 1:2000) and anti-β-actin (mouse, A5441; Sigma-Aldrich; dilution used in WB: 1:10000). Anti-SUV39H2 antibody (Sigma-Aldrich; dilution used in WB: 1:500) and anti-K392-dimethylated SUV39H2 antibody (Sigma-Aldrich; dilution used in WB: 1:1000 (Figure 3B), 1:250 (Figure 3C), 1:50 (Supplementary Figure S1)) were produced in rabbit immunized with a synthetic peptide.

### Mass spectrometry

The reaction mixture of *in vitro* methyltransferase assay was subjected to SDS-PAGE, and the bands on the gel were visualized by SimplyBlue™ SafeStain (Thermo Fisher Scientific). The bands corresponding to SUV39H2 were excised from the gel, and digested with sequencing grade TPCK-trypsin (Worthington Biochemical, Lakewood, NJ) and TPCK-chymotrypsin (Worthington Biochemical) in 30 μL of digestion buffer (10 mM Tris-HCl, 0.05% decyl glucoside, pH 8.0) at 37°C for 12 hours. The digest mixture was separated using a nanoflow LC (Easy nLC, Thermo Fisher Scientific) on an NTCC analytical column (C18, Φ0.075 × 100 mm, 3 μm, Nikkyo Technos, Tokyo, Japan) with a linear gradient of 35% buffer B (100% acetonitrile and 0.1% formic acid) at a flow rate of 300 nL/min over 10 min, and subjected on-line to a Q-Exactive mass spectrometer (Thermo Fisher Scientific) with a nanospray ion source using data dependent TOP10 method. The MS/MS spectra were searched against the in-house database using local MASCOT server (version 2.5; Matrix Sciences, London, UK). The quantitative analysis using Qual Browser (version2.2; Thermo Fisher Scientific) was performed as described previously [46]. The coverage ratio of this LC-MS/MS analysis was 90.6% (Supplementary Figure S2).

### *In vitro* methyltransferase assay

*In vitro* methyltransferase assays were performed as described previously [47–49]. Briefly, purified His-SUV39H2 expressed in Baculovirus infected insect cells or purified His-SUV39H2 with purified histone H3 expressed in *E.coli* or purified LSD1 expressed in Baculovirus infected insect cells was incubated in 50 mM Tris-HCl (pH 8.8) buffer with 1.0 μCi/ml S-adenosyl-L-[methyl-^3^H]-methionine (Perkin Elmer, Waltham, MA) for 2 hours at 30°C. After boiling in sample buffer, the samples were subjected to SDS-PAGE, and visualized by autoradiography [50].

### *In vivo* labeling

*In vivo* labeling was performed as described previously [11, 46]. 293T cells were starved for 0.5 hours in methionine-free medium, including cycloheximide (100 μg/ml) and chloramphenicol (40 μg/ml). They were then labeled with L-[methyl-^3^H] methionine (10 μCi/ml, Perkin Elmer) for 3 hours. Cell lysates co-overexpressing HA-LSD1 with FLAG-SUV39H2 WT, FLAG-SUV39H2 K392A, or FLAG-SUV39H2 K392R mutant were immunoprecipitated with HA agarose and methylated LSD1 was visualized by autoradiography.

### Immunoprecipitation

293T cells were seeded at a density of 40% on a 100-mm dish. After cell attachment, the cells were transfected with expression vectors using FuGENE™ 6 (Promega, Fitchburg, WI), and after 48 hours of incubation, transfected 293T cells were washed with PBS and lysed by RIPA buffer (50 mM Tris-Cl pH 7.4, 150 mM NaCl, 0.5% sodium deoxycholate, 0.1% SDS, 1% Nonidet-P40, 0.1 mM PMSF) with complete protease inhibitor cocktail (Roche Applied Science, Penzberg, Germany). Nuclear extracts were prepared to use in the experiment comparing the difference between wild-type SUV39H2 and mutant SUV39H2 (K392A and K392R). Cell extracts were incubated with anti-FLAG M2 affinity gel (Sigma-Aldrich) for 2 hours at 4°C. After the beads were washed 3 times with 1 ml of TBS buffer (pH 7.6), the FLAG-tagged proteins bound to the beads were eluted by boiling in Lane Marker Sample Buffer (Thermo Fisher Scientific). Samples were then subjected to SDS-PAGE, and detected by western blot.

## SUPPLEMENTARY FIGURES AND TABLE


