# Supplementary material for: Automethylation of SUV39H2, an oncogenic histone lysine methyltransferase, regulates its binding affinity to substrate proteins
Source: Oncotarget. 2016 Mar 14;7(16):22846–56. doi: 10.18632/oncotarget.8072 (PMC5008405; doi:10.18632/oncotarget.8072)
Supplement: Supplementary file 1 [file oncotarget-07-22846-s001.pdf]

SUPPLEMENTARY FIGURES AND TABLE

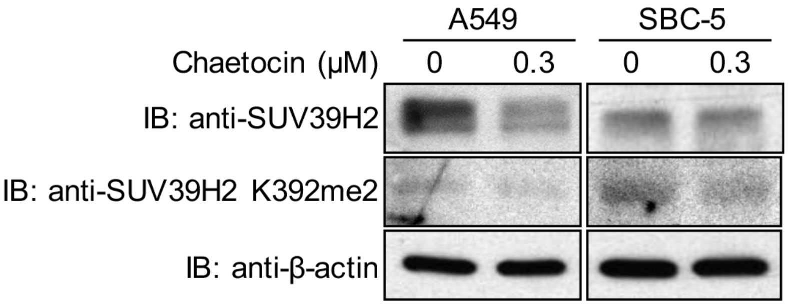

Supplementary Figure S1: The SU(VAR)3.9 specific inhibitor chaetocin attenuates automethylation of SUV39H2. 549 cells and SBC-5 cells were treated with 0 μM or 0.3 μM of chaetocin for 24 hours. Samples were immunoblotted with anti-SUV39H2, anti-SUV39H2 K392me2 and anti-β-actin antibodies.

1 MAAVGAEARG AWCVPCLVSL DTLQELCRKE KLTCKSIGIT KRNLNNYEVE

51 YLCDYKVVKD MEYYLVKWKG WPDSTNTWEP LQNLKCPLLL QQFSNDKHNY

101 LSQVKKGKAI T**PKDNNKTLK PAIAEYIVKK AKQRIALQRW QDELNRRKNH**

151 KGMIFVENTV DLEGPPSDFY YINEYKPAPG ISLVNEATFG CSCTDCFFQK

201 CCPAEAGVLL AYNKNQQIKI PPGTPIYECN SRCQCGPDCP NRIVQKGTQY

251 SLCIFRTSNG RGWGVKTLVK IKRMSFVMEY VGEVITSEEA ERRGQFYDNK

301 GITYLFDLDY ESDEFTVDAA RYGNVSHFVN HSCDPNLQVF NVFIDNLDTR

351 LPRIALFSTR TINAGEELTF DYQMKGSGDI SSDSIDHSPA KKRVRTVCKC

401 GAVTCRGYLN

Supplementary Figure S2: Amino acid sequence of SUV39H2, which is detected by LC-MS/MS analysis. Recombinant human His-tagged SUV39H2 (110aa – 410aa) was digested with trypsin and chymotrypsin, and red letters show the amino acid, which is detected by LC-MS/MS analysis. Among 299 amino acids in the recombinant protein, 271 amino acids were detected by this analysis (coverage ratio: 90.6%).

Supplementary Table S1: Information of certificated cell lines

Name	Certification institution	Tested method	DNA profile
**293T**	ATCC	STR	Amelogenin: X CSF1PO: 11, 12 D13S317: 12, 14 D16S539: 9, 13 D5S818: 8, 9 D7S820: 11 THO1: 7, 9.3 TPOX: 11 vWA: 16, 18, 19
**A549**	ATCC	STR	Amelogenin: X,Y CSF1PO: 10,12 D13S317: 11 D16S539: 11,12 D5S818: 11 D7S820: 8,11 THO1: 8,9.3 TPOX: 8,11 vWA: 14
**SBC-5**	JCRB	STR	Amelogenin:XY TPOX:9,12 CSF1PO:10 D5S818:10,11 D13S317:8,10 D7S820:8,11 D16S539:12 vWA:14,18 TH01:6

ATCC; American Type Culture Collection
JCRB; Japanese Collection of Research Bioresources
